# Data in support of the mutagenic potential of the isoflavone irilone in cultured V79 cells

**DOI:** 10.1016/j.dib.2015.07.010

**Published:** 2015-07-17

**Authors:** Anne Scheffler, Annette E. Albrecht, Harald L. Esch, Leane Lehmann

**Affiliations:** Institute of Pharmacy and Food Chemistry, Chair of Food Chemistry, University of Würzburg, Am Hubland, D-97074 Würzburg, Germany

## Abstract

The isoflavone irilone is found in human plasma after ingestion of red clover-based dietary supplements, but information allowing safety assessment is rare. Here, data in support of the mutagenic potential of irilone in cultured V79 cells [1] are presented. These data include (i) a quantitative assessment of irilone in the culture medium during the cell culture experiments, (ii) changes in the mutation spectrum in cDNA of the hypoxanthine-guanine phosphoribosyltransferase locus of irilone-treated V79 cells, (iii) occurrence of karyorrhexis and apoptosis as well as (iv) number of micronucleated cells containing whole chromosomes or chromosomal fragments. Also exemplary micrographs, used for the fluorescence microscopic assessment of (iii) and (iv) are presented.

**Table:** 

**Specifications Table**	
Subject area	Biology
More specific subject area	Toxicology, mutagenicity
Type of data	Table, figure, image (microscopy)
How data was acquired	DNA sequencing by Sanger method at LGC Genomics, Germany,Observer Z1 fluorescence microscope (Zeiss, Germany) equipped with a Plan-Apochromat objective 63x/1.4 Oil DIC
Data format	Raw (micronucleus data), analyzed (DNA sequencing, irilone in culture medium), and micrograph
Experimental factors	Cultured Chinese hamster V79 fibroblasts were treated with irilone. For cDNA sequencing total RNA was isolated from cloned mutants selected from irilone-treated, cultured Chinese hamster V79 fibroblasts, reversely transcribed, and amplified. For micronucleus data, cells were fixed, α-tubulin (Cy3), and centromere proteins (FITC) were stained immunochemically. DNA was stained with DAPI
Experimental features	Irilone in cell culture medium was quantified by HPLC-DAD. Mutation spectrum in cDNA of irilone-treated V79 cells was determined by Sanger method. Apoptosis, karyorrhexis, and micronucleus formation were assessed by fluorescence microscopy.
Data source location	
Data accessibility	Data are with this article only.

**Value of the data**

• Specifies mutation spectra and detailed discussion thereof.
• Complements data on mutagenicity, and cytotoxicity of irilone.
• Provides extensive raw data on time course of micronuclei formation observed with positive controls and irilone
• Methodical insight into scoring criteria for fluorescence microscopic evaluation

## 1 Data

### 1.1 Stability of irilone (IRI) under experimental incubation conditions

**Fig. 1:**
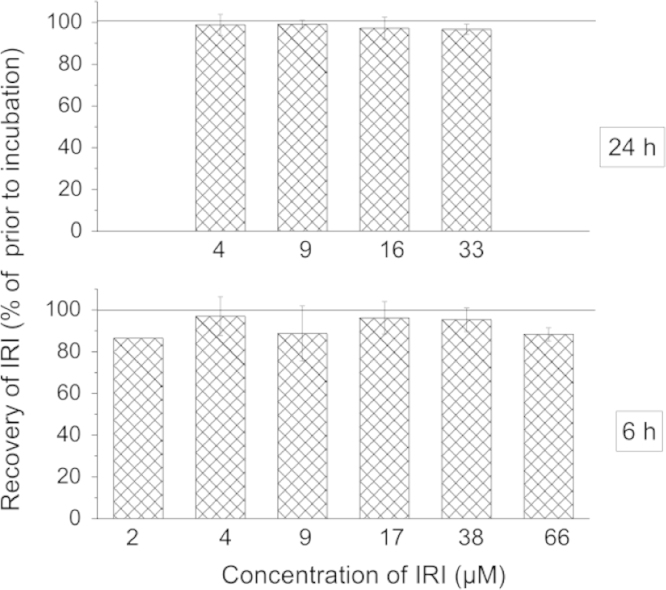
Recovery of IRI in cell culture medium after the incubation period in the hypoxanthine-guanine phosphoribosyltransferase (HPRT) assay (24 h, upper panel) and in the micronucleus test (6 h, lower panel). Prior to incubation, mean concentrations of IRI in medium and standard deviations of at least 3 independent experiments were 4.2±0.4 µM, 8.8±1.1 µM, 16.1±1.7 µM, and 32.7±2.1 μM in HPRT assays, and 2.2 µM (single experiment), 4.1±1.9 µM, 8.6±2.5 µM, 17.4±2.2 µM, 38.4±6.7 μM and 65.9±14.1 μM in micronucleus assays. In each experiment, the concentrations after incubation were related to the ones before incubation (100%). Data represent means±standard deviations of at least three independent experiments (6 h, 2 µM, single experiment). No significant differences between IRI concentrations before and after incubation were observed (*t*-test, adjusted *p*>0.05).

To evaluate the stability of IRI in cell culture medium under incubation conditions, aliquots of incubation media were either sampled immediately or at the end of the treatment period and concentrations of IRI were quantified by HPLC–UV: Concentrations of IRI in the culture media at the end of the treatment period of both the HPRT and micronucleus assay were not statistically different from that before treatment, yet mean values were slightly decreased (Fig. 1). Therefore, extensive chemical or enzyme-induced instability of IRI can be excluded.

### 1.2 Mutation spectra of IRI-induced V79 mutants

The mutation spectra of IRI-induced mutants formed during the hypoxanthine-guanine phosphoribosyltransferase (HPRT) assay in cultured V79 cells was investigated. Assuming that resistance of mutants to 6-TG has got to be reflected at *Hprt* mRNA level, the mutation spectra of *Hprt* cDNAs were determined. Thus, a minimum of ten 6-TG-resistant colonies derived from solvent- and IRI-treated cell populations were chosen randomly during each independent HPRT assay, cloned, total RNA was isolated, mRNA was reversely transcribed, cDNA was amplified by PCR, the amplification product purified, and sequenced by Sanger method. Thus, a total of 70 cDNAs, derived from 6-TG-resistant colonies of the populations treated with DMSO and 34 cDNAs, derived from populations treated with 16 µM IRI were analyzed. Each cDNA exhibited at least one mutation, i.e. deletions of exons 2–3, 4, 6, and of untranslated region+exons, transitions, and +1-frameshift mutations (Table 1). Mutation spectrum of 6-TG-resistant mutants derived from cells of the solvent control were characterized by exon deletions (67%) and base pair substitutions (33%, Table 1).

After treatment with 4 µM IRI the mutation spectrum of *Hprt* cDNAs exhibited one transition besides exon deletions (not observed in a total of 46 cDNAs obtained from populations treated with 16 and 33 µM IRI) and did not differ from that observed in control cells. In contrast, *Hprt* cDNA derived from cells treated with 33 µM IRI exhibited exclusively exon deletions and differed significantly from that observed in control *Hprt* cDNA (Table 1).

Comparison of spontaneously occurring mutation spectrum with published data was hindered due to methodical differences and variation between V79 cell lines: analysis of *Hprt* exons 1–9 of 30 and 40 DNAs derived from a V79 cell line originating in the same laboratory as the one used in the present study, revealed that exon deletions accounted for 40% [3] and 53% [4] of total mutations, respectively. However, at cDNA level, deletions are not only caused by exon deletions but also by base pair substitution(s), frameshift mutations and other events [5].

Thus, the proportion of exon deletions at cDNA level observed in the present study (67%) is probably lower at DNA level and thus comparable or even lower than that observed at DNA level with the same V9 MZ, yet higher than that determined at cDNA level in a V79 cell line of unknown origin. Since no mutants with deletions in one or more exons of Hprt DNA were observed in the V79-UL cell population, which is also a “normal” V79 cell line from another laboratory [3,4], big variances between different V79 cell lines can be expected. It is known that also the response to mutagens differs between “normal” V79 cell populations [6]. Thus, it can only be stated that the mutation spectrum of the DMSO-treated, 6-TG resistant V79 cell population in the present study seems not to be unusual for the V79 MZ population.

The determination of the mutation spectrum had two limitations:

(i) two or more identical mutations at the same position of *Hprt* cDNA were observed in mutants selected from the same flask. Therefore, mutations were only assumed to be independent, if they were either at different positions of the *Hprt* cDNA sequence, occurred in different experiments, which were performed with a freshly thawed stock of cells, or occurred in cell populations treated in different flasks during the same HPRT assay. As a consequence, despite sequencing 70 cDNAs derived from control cell populations and 34 cDNAs derived from populations treated with IRI, the number of independent mutations for which clonal expansion could be completely excluded was small (Table 1). Yet, the effect was still obvious without any statistical analysis and reached statistical significance despite the rather small number of observations. Independency of mutations observed *in vitro* is defined and dealt with heterogeneously in literature: (a) mentioning the possibility of clonal expansion in discussion but ignoring it in data analysis (e.g. [7]), (b) defining mutations to be independent if picked from different petri dishes seeded from the same cell solution (e.g. [8]), (c) same as (b) and repeating the experiment once, e.g. [3], (d) splitting the culture after treatment into different flasks (e.g. [9]). Thus, the authors’ approach was the most conservative one possible, comparable to that of [9].
(ii) *Hprt* cDNAs instead of *Hprt* exons were sequenced for assessment of mutation spectra. However, base pair substitutions, frameshift mutations, and deletions in *Hprt* introns and exons may cause the same results on cDNA level [5]. Thus, information on the genotoxic mode of action derived from analysis of *Hprt* cDNA was not as detailed as the one that would have been derived from analysis of *Hprt* exons, and unambiguous information could only be achieved by analysis of both *Hprt* exons and introns together. Due to the higher information content, it can be assumed that analysis of DNA would detect a shift in mutation spectrum rather more sensitively than analysis of cDNA. Thus, the observed IRI-induced alteration of the mutation spectrum at cDNA level can be assumed to be confirmed at DNA level. However, the multiple causes possibly leading to the observed increase in the proportion of cDNA deletions induced by IRI [1], would render a detailed discussion of the putative genotoxic mode of action of IRI and of the cause for the lack of increase in mutant frequency rather speculative.

Although exon deletions at cDNA level are caused by both deletions, base pair substitutions, and frameshift mutations at DNA level [5], the increase in exon deletions in mutation spectra of 6-TG resistant V79 cells indicated a clastogenic mode of action, which is known to be detected insufficiently by the *Hprt* gene mutation assay [10]. Thus, the putatively clastogenic potential of IRI was further investigated using the micronucleus assay, which responds to both clastogens inducing micronuclei containing chromosomal fragments (CREST antibody signal-negative micronuclei) and aneugens inducing micronuclei containing whole chromosomes (CREST antibody signal-positive).

### 1.3 Fluorescence microscopic assessment of cytotoxicity, apoptosis, and micronuclei formation

**Fig. 2:**
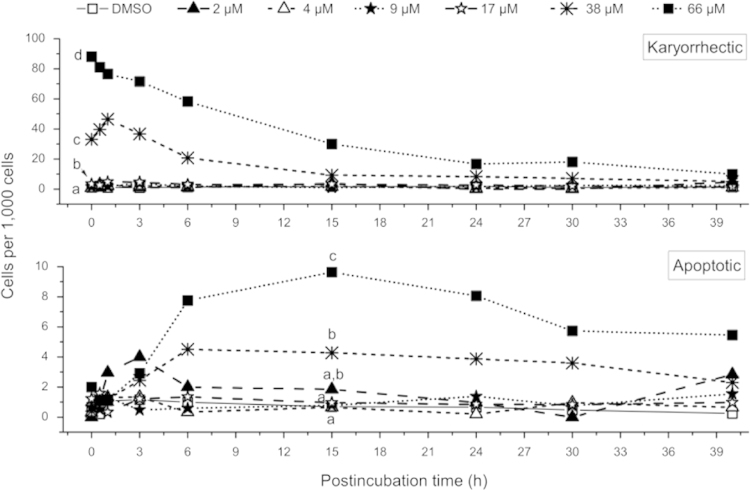
Number of cells exhibiting nuclear fragmentation without (i.e. karyorrhectic, top panel) and with chromatin condensation (i.e. apoptotic, bottom panel) in V79 cells immediately after treatment with IRI for 6 h and subsequent IRI-free postincubation for up to 40 h. Data represent sums of minimum three independent experiments, statistically analyzed by Fisher’s exact test. Different characters signify statistically different data groups at the respective postincubation time (*p*<0.05). The number of karyorrhectic cells in the population of cells treated with 38 and 66 µM IRI stayed statistically different from control populations until postincubation for 40 h (information not included in the graph).

As expected, no significant increase in the frequency of cells containing nuclear fragments with condensed chromatin, i.e. apoptotic bodies (Fig. 2), was observed immediately after treatment with IRI for 6 h (Fig. 2). After IRI-free postincubation for up to 40 h, small increases in the frequencies of cells with apoptotic bodies were observed after treatment with 38 and 66 µM IRI (maximum 4 and 10 cells/1000 cells, respectively, at 15 h postincubation, Fig. 2). However, after treatment with 17, 38, and 66 µM IRI, a strong increase in the frequency of nuclear fragmentation (karyorrhexis) without chromatin condensation was observed, decreasing during postincubation time and remaining statistically significant until 6 h (38 µM) and 15 h (66 µM) of postincubation (Fig. 2).

In the V79 cell population treated with solvent only, 20–34 cells containing CREST antibody signal-negative micronuclei (i.e. chromosomal fragments)/3000 cells were observed (Supplemental Table, and [1]) which lies within the normal range [11]. The aneugen diethylstilbestrol (DES) did not affect the frequency of micronucleated cells with chromosomal fragments at any time point (Supplemental Table). The occurrence of micronuclei containing chromosomal fragments induced by other positive controls followed different kinetics: whereas the topoisomerase poison etoposide significantly increased the frequency of cells with CREST antibody signal-negative micronuclei already at 6 h postincubation time, the DNA-DNA cross-linker mitomycin C and the alkylating agent ethyl methanesulfonate induced an increase in micronucleated cells at 15 and 24 h, respectively. In cell populations treated with 38 and 66 µM IRI, a slight but significant (*p*<0.05, Fisher’s exact test) increase in the frequencies of micronucleated cells containing chromosomal fragments at 6–40 h (38 and 66 µM) postincubation time to maximum 53 and 64 micronucleated cells/3000 cells, respectively, was observed, supporting a weak clastogenic potential of IRI. Although the increase in micronucleated cells was too small to draw strong conclusions from its time-dependency, a mode of action needing rather a shorter postincubation period (such as observed with etoposide or mitomycin C) than a longer postincubation period (as observed with ethyl methanesulfonate) was indicated (Supplemental Table, and [1]).

After treatment with the known aneugen diethylstilbestrol (15 and 25 µM), the frequency of cells with micronuclei containing whole chromosomes at 0–15 h postincubation time was increased to maximum 89 micronucleated cells/3000 cells (25 µM, 6 h postincubation) compared to 2.6–6.1 micronucleated cells/3000 cells observed with the solvent control (Supplemental Table, and [1]). Likewise, after treatment with 38 and 66 µM IRI, the frequency of micronucleated cells was increased to maximum 40 (38 µM, 3 h postincubation) and 41 (66 µM, 6 h postincubation) micronucleated cells/3000 cells (Supplemental Table, and [1]).

## 2 Experimental designs, material and methods

### 2.1 General cell culture conditions

V79 MZ cells, kindly provided by H. Glatt (German Institute of Human Nutrition, Potsdam, Germany), were cultured in DMEM supplemented with 100 U/ml penicillin, 100 μg/ml streptomycin, and 10% fetal calf serum (Invitrogen™ Life Technologies, Germany) referred to as DMEM complete. Cells were kept at 37 °C in a humidified atmosphere and 5% CO_2_.

### 2.2 Quantitation of IRI in cell culture medium before and after incubation

In each experiment, about 1 ml of the incubation medium (freshly prepared and after incubation) was flash frozen with liquid nitrogen and stored at −80 °C until analysis. To quantify IRI, the method previously described for the determination of IRI in dietary supplements [12] was used with slight modifications. 2.0 µg internal standard (4,4′-isopropylidendiphenol) was added to 250 µl (<10 µM IRI) or 50 µl (>10 µM IRI) medium prior to extraction with ethyl acetate. Organic phases were collected, evaporated, and the remainder was reconstituted in 50 µl methanol. HPLC analyses were run on a HP Agilent Series 1100 (Agilent Technologies, Germany) equipped with a diode array detector. 5 µl reconstituted extract was separated on a C18 phase (Eurospher-100, 250×4 mm, 5 μm, Knauer, Germany) at 1 ml/min using acidified acetonitrile:water (0.1% formic acid in water (A) and 0.1% formic acid in acetonitrile (B); gradient was from 30% B to 39% B over 54 min and then from 39% B to 55% B over 16 min), detected by its absorbance at 280 nm and relative peak areas compared to the calibration curve (24, 44, 64, 84, and 104 ng IRI together with 200 ng internal standard on column).

### 2.3 Mutation spectrum of IRI treated V79 cells

#### 2.3.1 HPRT assay for the generation of mutants

HPRT assays have been performed as described previously [13] with slight modifications. 1.5×10^6^ V79 cells were seeded in cell culture flasks (175 cm^2^, Greiner Bio-one, Germany) containing 20 ml DMEM complete. After 24 h, the medium was changed (day 0) and cells were treated with different concentrations of IRI or 1 µM NQO or 1% DMSO for 24 h. A total of 1×10^6^ treated cells were subcultured in fresh medium directly after treatment (day 1) and again on day 4. On day 6, cells with mutations at the *Hprt* gene locus were selected by growing cells in DMEM complete, and 7 µg 6-thioguanine (6-TG)/ml using three tissue culture dishes (145 mm, Greiner Bio-one) with 1×10^6^ cells per dish.

#### 2.3.2 Cloning of 6-TG-resistant mutants

After 12 days, medium from the dishes containing the *Hprt* mutants was removed and single *Hprt* mutant colonies were trypsinized within sterilized stainless steel rings, placed around single colonies using silicone grease. Detached cells were transferred into single wells of 6-well plates (Nunc^TM^, Thermo Fisher Scientific, Germany) and cultured for another 2–4 days until isolation of total RNA.

#### 2.3.3 RNA isolation and reverse transcription

Total RNA was isolated using the GenElute™ Mammalian Total RNA Miniprep Kit followed by DNase digestion by DNase I Amplification Grade Kit (both kits Sigma-Aldrich) and stored at −80 °C. One µg total RNA was reversely transcribed using oligo-(dT)18 primer, 10 mM dNTPs, 20 u RiboLockTM RNase-Inhibitor, and 200 U RevertAidTM Reverse Transcriptase according to the manufacturer’s instructions (Thermo Scientific, Germany). cDNA was stored at −20 °C.

#### 2.3.4 Amplification, isolation and sequencing of V79 cDNA

**Fig. 3:**
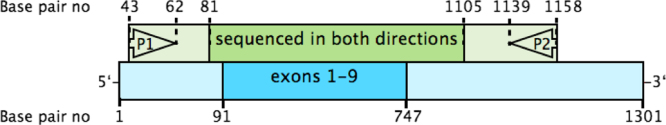
*Hprt* primer positions, amplified and sequenced sequences of *Hprt* cDNA. Primers (P1 and P2) were positioned in the 3′- and 5′-untranslated region (light blue) of *Hprt* cDNA (reference cDNA sequence: GenBank: J00060.1; http://www.ncbi.nlm.nih.gov/nuccore/J00060.1) 29 base pairs distant from exon 1 (P1) and 392 base pairs distant from exon 9 (P2). The first few base pairs cannot be analyzed reliably by Sanger sequencing (light green) but base pairs 81 and higher as well as 1105 and lower were analyzable in every sequencing reaction (green). (For interpretation of the references to color in this figure legend, the reader is referred to the web version of this article.)

Reverse primer 5′-ATGAACTGAGTGCTTTCACA-3′ (exact positions depicted in Fig. 3) and forward primer 5′-CTTCCTCCTCACACCGCTCT-3′ were checked for lack of secondary structures and unspecific amplification products by PrimerExpress 3.0 – licensed – (Applied Biosystems). Positioning of primers in the 3′- and 5′-untranslated region of *Hprt* cDNA, respectively, ensured reliable sequencing and duplicate determination of all exons (Fig. 3). 600 ng *Hprt* cDNA dissolved in nuclease free water and 4 μl forward or reverse primer (5 μM), respectively were mixed in a volume of 14 µl and subjected to Sanger sequencing by LGC Genomics, Germany.

#### 2.3.5 Analysis of sequencing data and of mutational spectra

Resulting electropherograms were analyzed using Chromas Lite (freely available at http://www.softpedia.com/get/Science-CAD/Chromas-Lite.shtml) and resulting sequences were compared using ApE (freely available at http://biologylabs.utah.edu/jorgensen/wayned/ape/) and Referenz Sequenz cDNA: GenBank: J00060.1; http://www.ncbi.nlm.nih.gov/nuccore/J00060.1. Type and position of mutations within the cDNA were determined by sequence alignment.

### 2.4 Micronucleus assay

#### 2.4.1 Cell exposure

V79 cells were grown on chamber slides (Thermo Fisher Scientific Inc., Germany) with eight chambers per slide (5–7×10^3^ cells/cm^2^) for 24 h prior to incubation with IRI (ChromaDex™, Irvine, CA, USA), diethylstilbestrol (Biomol, Germany), etoposide, mitomycin C, and ethyl methanesulfonate (all Sigma-Aldrich, Germany) or with the solvent (1% DMSO) for 6 h with and without subsequent substance-free incubation for up to 40 h.

#### 2.4.2 Fixation and immunological staining

Following incubation with the test compounds, cells were stained as described previously [14] with slight modifications. Cells were fixed with freshly depolymerized 3.5% paraformaldehyde in enriched PBS pH 6.1 for 1 min at room temperature and methanol at −18 °C for at least 1 h. Non-specific binding of antibodies was blocked by incubation with goat serum (Sigma-Aldrich) for 1 h at 37 °C. Cells were stained with anti-α-tubulin (Sigma-Aldrich, 1:500 in 1% bovine serum albumin containing PBS) for the evaluation of cell morphology and the detection of freshly divided cells, and with anti-centromere protein antibodies (from patients with limited systemic scleroderma/CREST syndrome, Antibodies Inc., Davis, CA, USA, 1:100 in 1% bovine serum albumin containing PBS) followed by secondary Cy3-conjugated goat anti-mouse antibody (Jackson Immune Research Laboratories, Inc., West Grove, USA, diluted 1:250 in PBS) and FITC-conjugated goat polyvalent anti-human antibody (Sigma-Aldrich, diluted 1:148 in PBS) for 1 h at 37 °C each. Finally, slides were mounted with antifade solution containing 1 µg 4′,6-diamidino-2-phenylindole (DAPI)/ml.

#### 2.4.3 Fluorescence microscopic analysis

Slides were coded and analyzed with an Observer Z1 fluorescence microscope (Zeiss, Germany) equipped with a Plan-Apochromat objective 63x/1.4 Oil DIC by a person not knowing the code. Scoring criteria for micronuclei [15] were considered, supplemented by the following: nucleus and micronucleus/micronuclei were to lie within the same cytoskeleton (i.e. α-tubulin staining). Moreover, normal and disrupted mitotic stages, freshly divided cells (i.e. cells with cytoplasmic bridges) as well as nuclear fragmentation and apoptotic cells were scored (see exemplary micrographs next section).

On each slide, nuclei and micronuclei were visualized using ultraviolet excitation (characteristics of excitation filter, beam splitter, and emission filter were EX G 365, BS FT 395, EM BP 445/50, respectively) and the microtubules (forming the cytoplasmic microtubule complex, the midbody and the mitotic spindle) and signals of centromere proteins were analyzed under green (EX BP 550/25, BS FT 570, EM BP 605/70) and blue (EX BP 470/40, BS FT 495, EM BP 525/50) excitation, respectively. All signals were visualized simultaneously by means of a triple bandpass filter (EX TBP 406+489+561; BS TFT 427+503+578; EM TBP 459+525+608). At least 1000 cells or 100 cells with micronuclei were scored per slide with respect to micronuclei, morphology of mitosis, cell divisions and signs of cytotoxicity such as nuclear fragmentation, apoptotic bodies, and depolymerized cytoplasmic microtubule complex.

#### 2.4.4 Exemplary micrographs of endpoints scored microscopically

Micrographs were taken using AxioCam MRm3 S/N 5925 digital camera controlled by ZEN pro 2011 software (Zeiss, Germany). Each event was photographed with the optical filter set specific for DAPI fluorescence of DNA, Cy3 fluorescence of α-tubulin, and, in some cases, FITC fluorescence of centromere proteins, using 37 Z-Stacks (i.e. focal planes). Exposure time was set individually to use maximum dynamic range indicated by the histogram. Calibration bars were added to original.czi files using *Insert Scale Ba*r function of the ZEN desk 2012 software (Zeiss). Then, Z-Stacks were reduced to one plane by the algorithm *Extended Depth of Focus* using the variants *Contrast*, *Wavelets* or *Variance* as appropriate. Alternatively, a single *Z*-Plane was chosen. If image sections were needed, they were generated using *Create Image Subset*.

To generate overlays, micrographs of the same event taken with different optical filter sets, were merged using *Add Channels*. Channels were colored individually light blue (DNA), orange/red (α-tubulin), and – when applicable – green (centromere proteins).

***Normal interphase cells***

**Figure:**
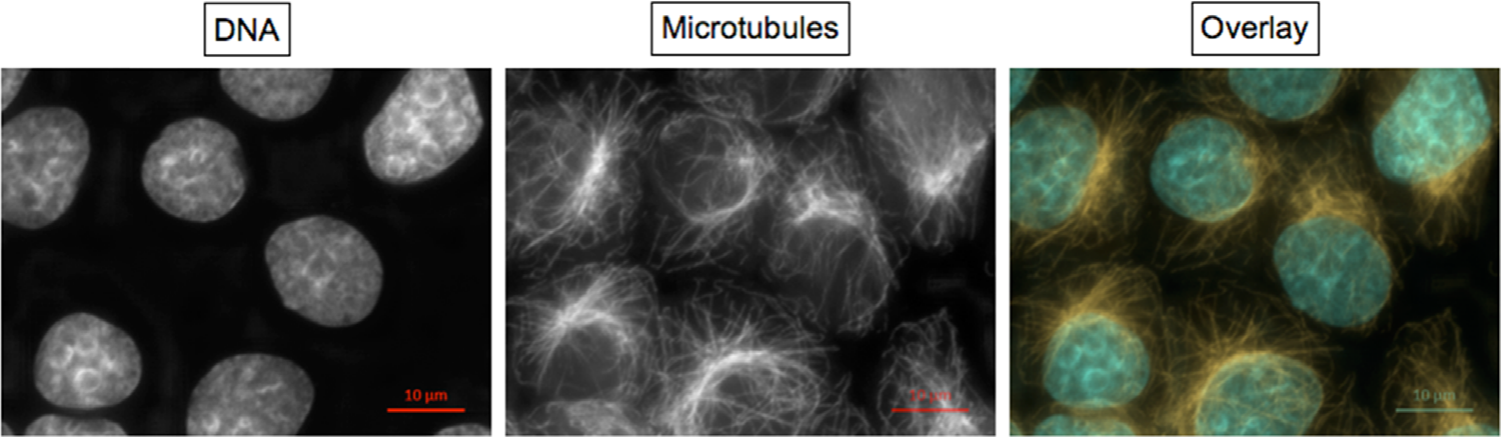

Each nucleus lies within a single cytoplasmic microtubule complex (CMTC). Intact microtubuli are oriented from the microtubule organizing center (usually indicated by the highest fluorescence intensity of α-tubulin staining within the CMTC) towards the cell membrane.

***Nuclear fragmentation without and with chromatin condensation***

**Figure:**
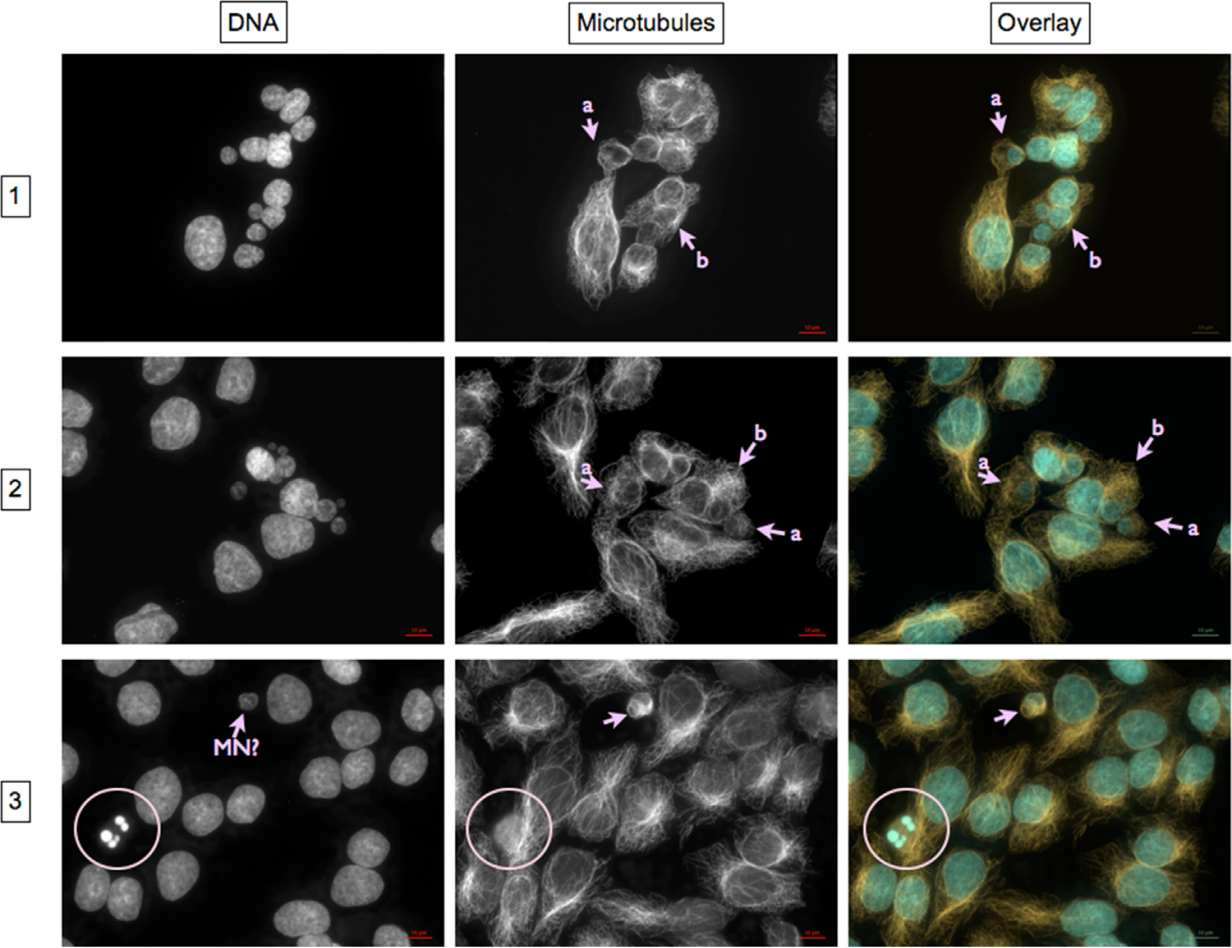

Panel 1 and 2: One (a) or multiple (b) nuclear fragments without chromatin condensation lie within an intact CMTC. In the latter case, no apparent main nucleus is discernible.

Panel 3: Besides fragmentation of the nucleus, apoptosis is indicated by condensed chromatin (bright DAPI staining of DNA, encircled) and depolymerized microtubules (encircled). DAPI fluorescence indicates a micronucleus (MN?), which is disproved by lack of a main nucleus within the same cytoplasmic microtubule complex.

***Micronucleated cells***

**Figure:**
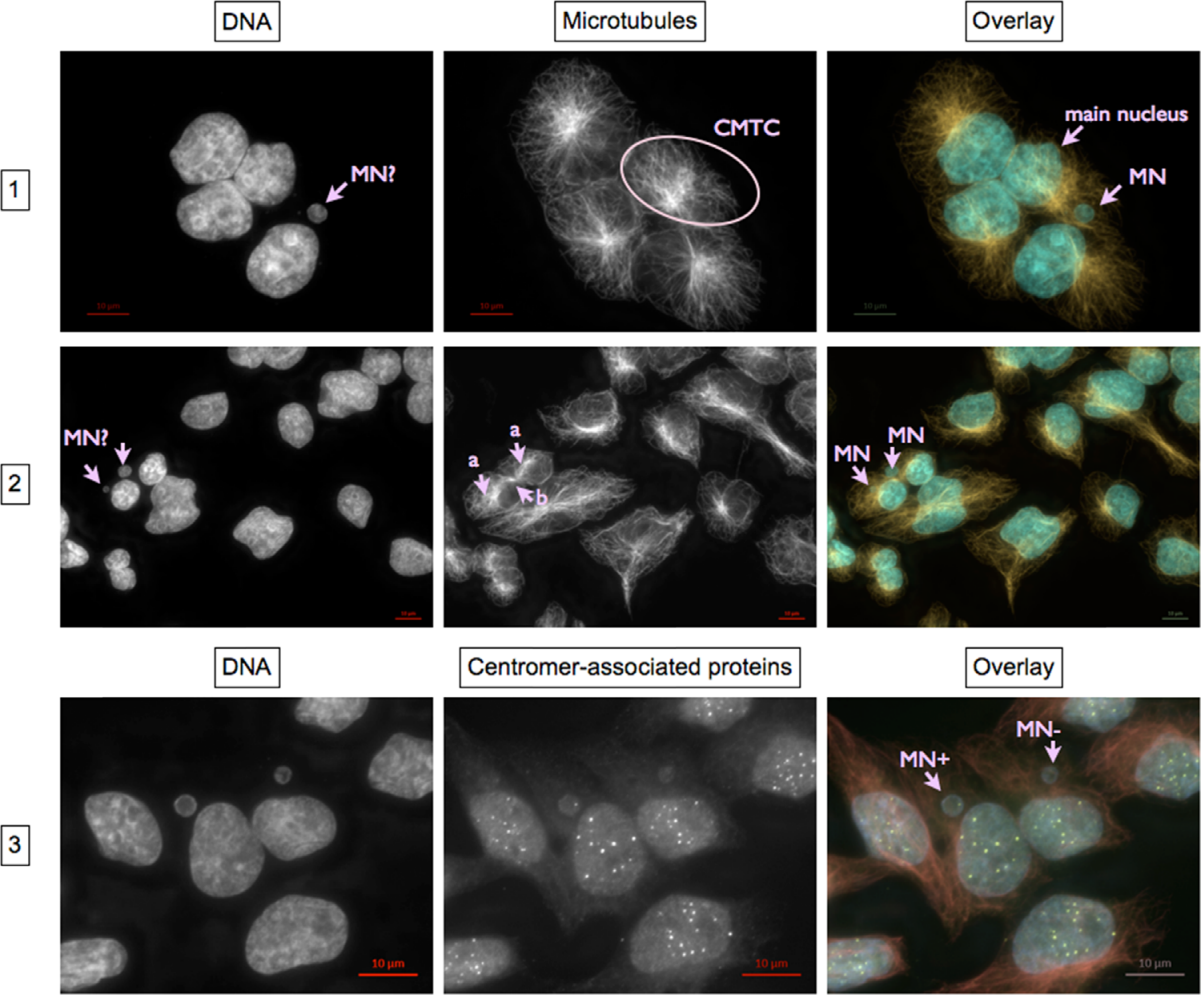

• Panel 1: The micronucleus (MN) lies together with an obvious main nucleus within the CMTC of a single cell indicated by a single microtubule organizing center.
• Panel 2: Remnants of the midbody identify daughter nuclei formed during a recent cytokinesis. Thus, each of the two micronuclei visible belongs to one of the two daughter cells.
• Panel 3: Signals of immunochemically stained centromere proteins identify MN containing whole chromosomes. Thus in panel 3, the MN on the left contains a whole chromosome (MN+) and the MN on the right contains a chromosomal fragment (MN-).

***Normal mitotic stages and interphase cells***

**Figure:**
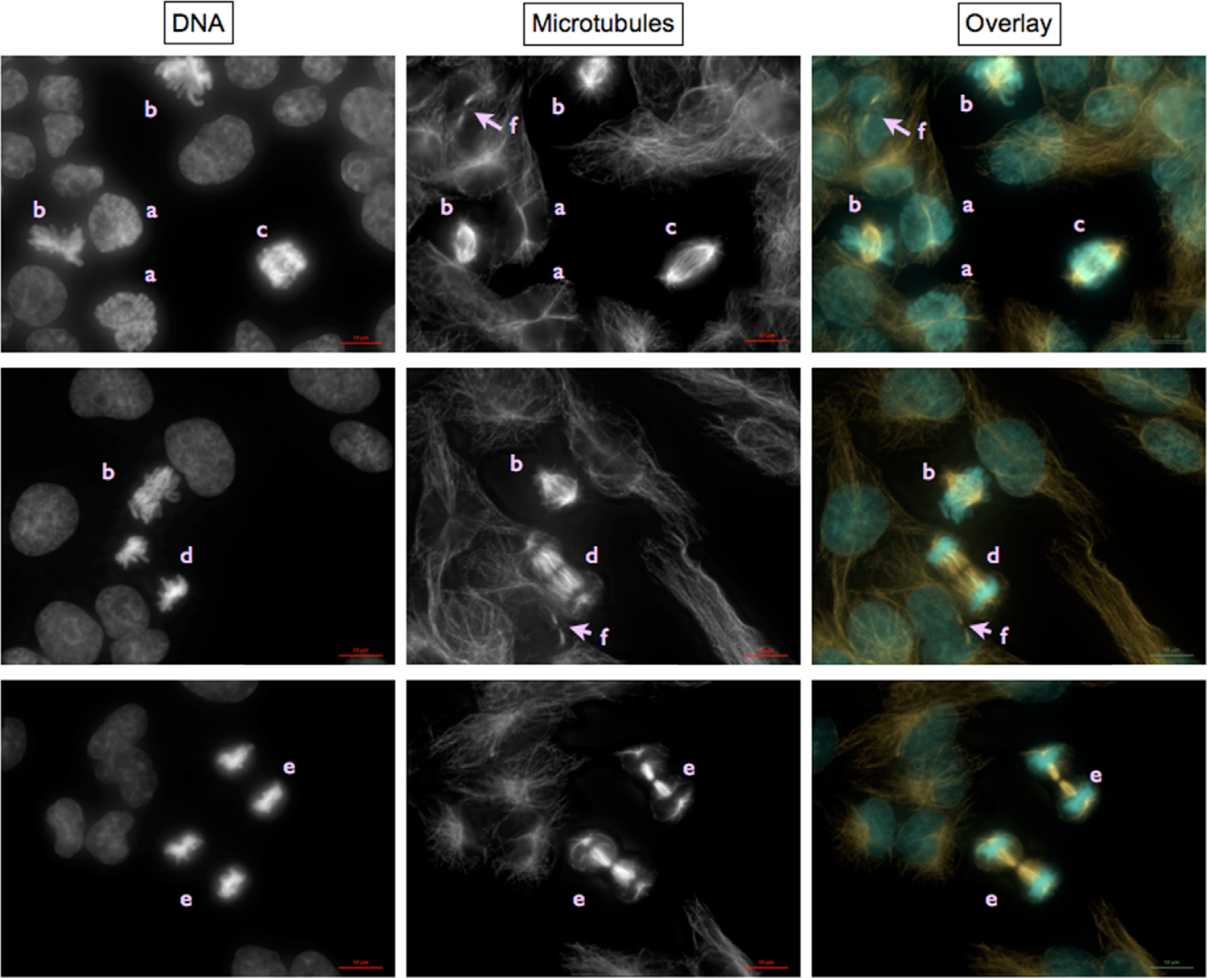

*a*—prophase; *b*—metaphase; c—anaphase I; d—anaphase II; e—telophase; f—two daughter cells formed during a recent cell division identified by remnants of the midbody indicating position of cytokinesis.

***Disrupted mitotic stages***

Metaphases with disrupted mitotic spindles

**Figure:**
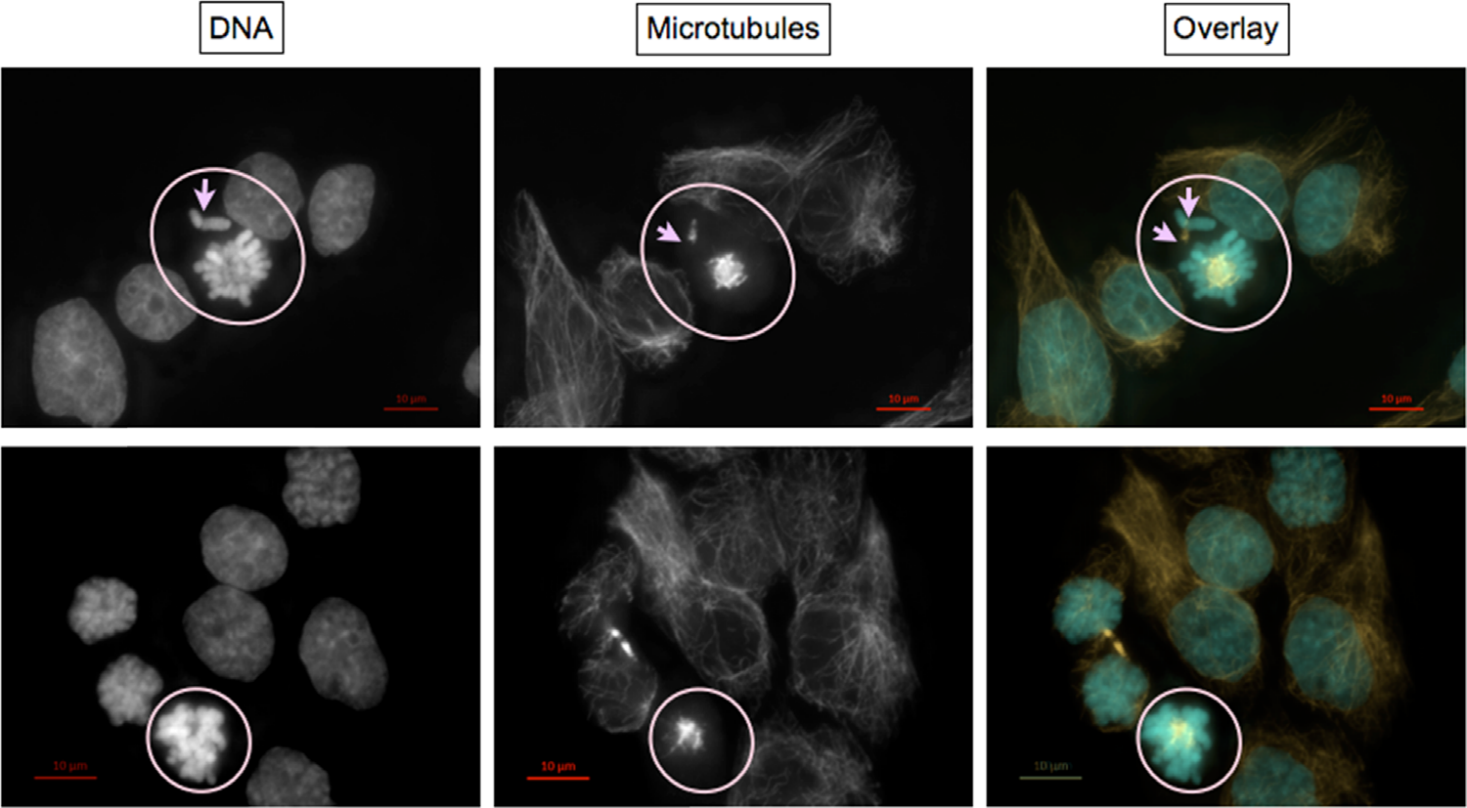

Encircled mitotic spindles contain less and/or shorter and/or disorganized microtubules and/or are monopolar; most likely leading to polyploidy or mitotic catastrophe. More examples are visible in the micrographs shown at “Asymmetric cell divisions…” panels 2, 4, and 5, circles c.

Metaphases with tri- and multipolar mitotic spindles

**Figure:**
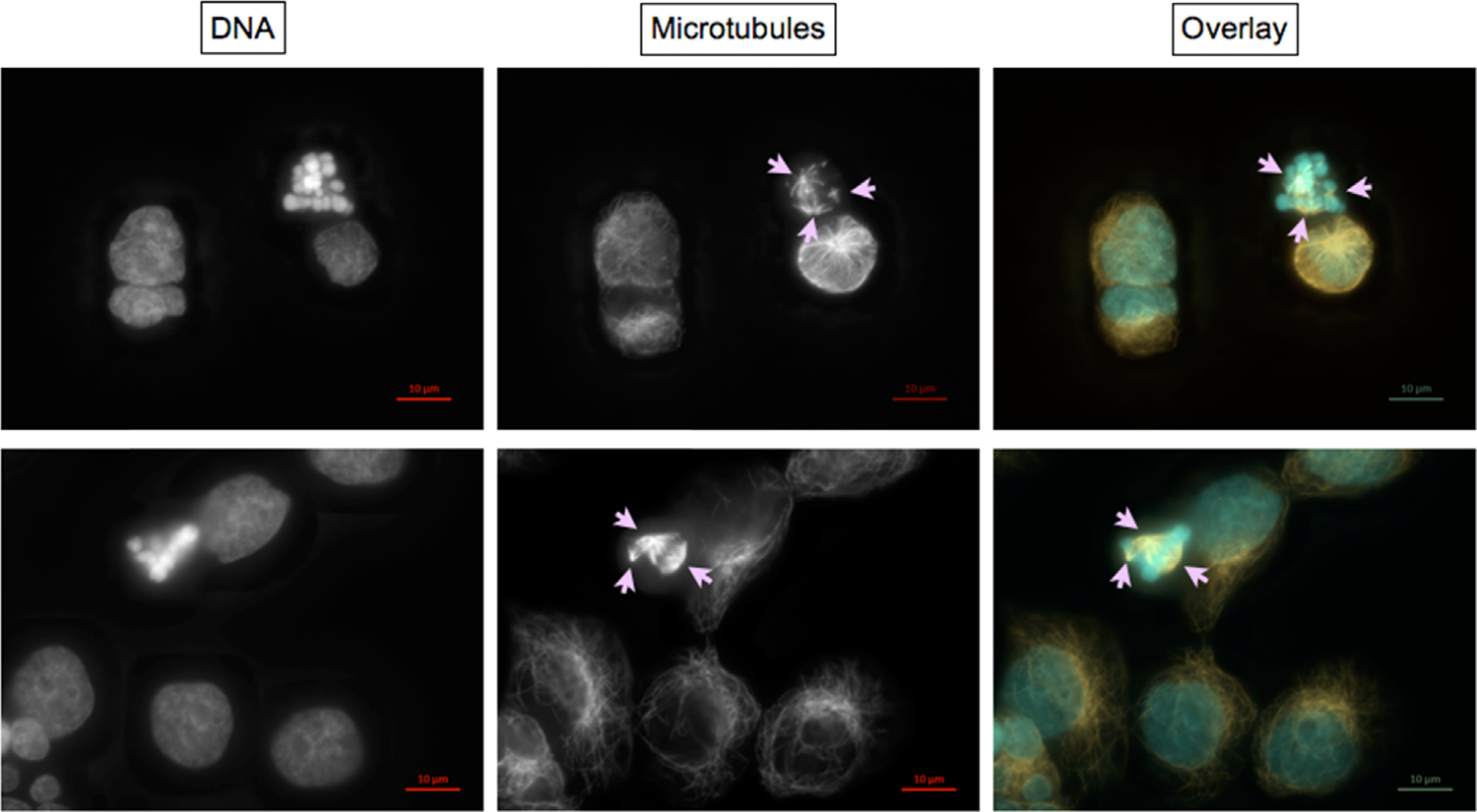

Chromosomes are oriented between three or more spindle poles (arrows).

Asymmetric cell divisions caused by ana- and telophases with tri- and multipolar mitotic spindles and lagging chromosomes

**Figure:**
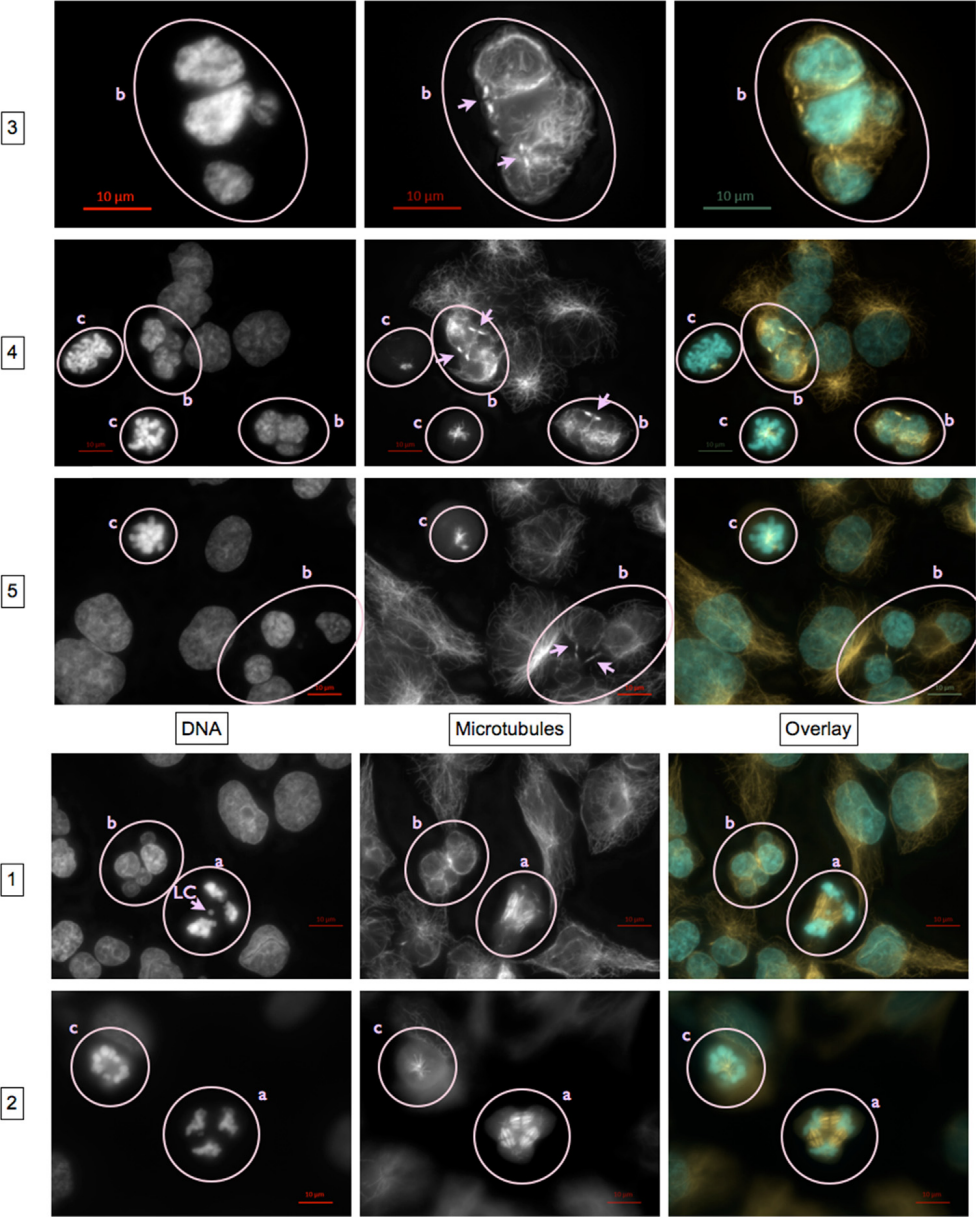

Panels 1 and 2, circles a:

Chromosomes are oriented between three or more spindle poles. Depending on the distribution of the other chromosomes, lagging chromosomes (LC) might appear as one of several “nuclei” distributed evenly or unevenly between two or more daughter cells, in some cases meeting the criteria for micronuclei.

Panels 1, 3, 4, and 5, circles b:

Possible results of asymmetrical mitotic spindle assembly. Chromatin is distributed unevenly between several “nuclei” of different sizes which lie within 2 to 3 daughter cells as identified by remnants of the midbody indicating position of previous cytokinesis (arrows).

Panels 2, 4, and 5 circle c:

Disrupted mitotic spindles in (pro-)metaphase.

## Figures and Tables

**Table 1:** Mutation spectra of *Hprt* cDNAs derived from 6-thioguanine (6-TG)-resistant colonies selected from V79 cell populations treated with solvent (1% DMSO) or IRI. In each independent HPRT experiment (1, 2, 3), two flasks were treated with solvent (DMSO1 and DMSO2) and one flask each with various concentrations of IRI. The change in the sequence of cDNA is given in 5′→3′direction. Despite sufficient amount and quality of total RNA, 23% of *Hprt* cDNAs collected after treatment with DMSO yielded no PCR product, which did not differ statistically from the percentage observed after treatment with 16 µM IRI (18%, *p*=0.6166, Fischer’s exact test). Since the primer binding sites were in the untranslated regions (UTR) flanking exons 1–9 and the mutant clone was 6-TG-resistant, no PCR product can only be due to complete lack of *Hprt* cDNA or deletions affecting one or more exons together with one or both primer binding sites. Thus, mutants not yielding a PCR product were scored “deletions of UTR+exons (UTR+?)”. The same mutation at the same locus in cDNA extracted from colonies of the same V79 cell population (i.e. one flask) may be due to the same event and were therefore counted only once for the determination of the spectra of certain independent mutations (*Σi*). In the table, each independent mutation is highlighted by a grey background, thus the number of grey rectangles per treatment group corresponds to *Σi*. Statistical comparison of complete mutation spectra (*Σ*, *Σi*) was performed according to [2].

**Position**		**Change in sequence**		**Number of mutations**										
**Exon**	**Bp**	**cDNA**	**Amino acid**	**DMSO1**			**DMSO2**			**IRI 16** µ**M**			**IRI 4** **µM**	**IRI 33** **µM**
				**1**	**2**	**3**	**1**	**2**	**3**	**1**	**2**	**3**	**3**	**3**
1	81	ACC→ACG	Thr→Thr	0	0	0	0	1	0	0	0	0	0	0
3	241	CGA→TGA	Arg→Stop	0	0	0	0	0	0	0	0	0	1	0
6	493	GAT→AAT	Asp→Asn	0	5	2	0	0	2	0	0	0	0	0
6	499	ATT→TTT	Ile→Phe	0	0	0	2	0	0	0	0	0	0	0
6	505	ACT→CCT	Thr→Pro	1	0	0	0	0	0	0	0	0	0	0
7	584	GTG→GGG	Val→Gly	0	0	0	0	4	0	0	0	0	0	0
8	631	TTT→ATT	Phe→Ile	0	0	0	1	0	0	0	0	0	0	0
8	662	TAT→TGT	Tyr→Cys	0	0	0	3	0	0	0	0	0	0	0
8	681	GAG→GAC	Glu→Asp	1	0	0	0	0	0	0	0	0	0	0
9	707	TGT→TAT	Cys→Tyr	0	0	0	1	0	0	0	0	0	0	0
3	405/406	Insertion C		0	0	0	0	0	0	1	0	0	0	0
8/9	699/700	Insertion G		0	0	0	0	0	0	0	1	0	0	0
2+3	109–409	Deletion	Deletion	0	0	0	0	0	2	0	3	0	0	0
2	110–225	Deletion	Deletion	00	0	0	0	0	0	1	0	0	0	0
4	413–478	Deletion	Deletion	7	0	9	2	3	5	10	7	5	8	7
6	493–575	Deletion	Deletion	0	0	0	0	0	3	0	0	0	0	0
UTR+?	?	Deletion	Deletion	3	7	1	3	2	0	0	1	5	3	5
				*Σ*36 (*Σi* 9)			*Σ*34 (*Σi* 14)			*Σ*34 (*Σi* 9)			*Σ*12 (*Σi* 3)	*Σ*12 (*Σi* 2)
*p* Value of comparison *Σ* treatment group vs. *Σ* DMSO1+2				0.67059			0.84118			0.02941			0.357060	0.70177
*p* Value of comparison *Σi* treatment group vs. *Σi* DMSO1+2				1.00000			1.00000			0.66353			–	–
