# Supplementary material for: Data in support of the mutagenic potential of the isoflavone irilone in cultured V79 cells
Source: Data Brief. 2015 Jul 17;4:474–87. doi: 10.1016/j.dib.2015.07.010 (PMC4534604; doi:10.1016/j.dib.2015.07.010)
Supplement: Supplementary file 1 — Supplementary data [file mmc1.docx]

**Supplemental Table: Micronucleated cells scored in the single experiments of the micronucleus assay**

In each individual experiment 1,000-2,000 cells were scored. For better comparison between the single experiments, the numbers of micronucleated cells containing chromosomal fragments (MN-) and whole chromosomes (MN+) per 1,000 cells are given, supplemented with the total number of cells scored (∑cells scored).

The time points indicate period of incubation (6 h) + period of compound-free postincubation (0-40 h). -, not determined.

|  | **Micronucleated cells per 1,000 cells** | | | | | | | | | | | | **∑cells scored** |
| --- | --- | --- | --- | --- | --- | --- | --- | --- | --- | --- | --- | --- | --- |
|  | **MNC-** | | | | | | **MNC+** | | | | | |  |
|  | Experiment | | | | | | Experiment | | | | | |  |
|  | 1 | 2 | 3 | 4 | 5 | 6 | 1 | 2 | 3 | 4 | 5 | 6 |  |
| **6+0 h** | | | | | | | | | | | | | |
| **0.1 % DMSO** | 11 | 10 | 12 | 8 | 9 | 15 | 0 | 0 | 1 | 2 | 1 | 1 | **10,328** |
| **IRI 2 µM** | - | - | - | 10 | - | - | - | - | - | 2 | - | - | **1,088** |
| **IRI 4 µM** | - | - | - | 9 | 9 | 8 | - | - | - | 1 | 3 | 1 | **3,159** |
| **IRI 9 µM** | 5 | 8 | 5 | 7 | - | - | 3 | 1 | 5 | 3 | - | - | **5,039** |
| **IRI 17 µM** | 7 | 8 | 8 | - | - | - | 4 | 2 | 4 | - | - | - | **3,112** |
| **IRI 38 µM** | 12 | 18 | 7 | 11 | - | - | 5 | 8 | 10 | 18 | - | - | **8,25** |
| **IRI 66 µM** | 6 | 8 | 6 | 14 | - | - | 6 | 9 | 8 | 9 | - | - | **8,121** |
| **DES 15 µM** | - | - | - | 9 | 5 | - | - | - | - | 11 | 9 | - | **2,133** |
| **DES 25 µM** | 3 | 10 | 5 | - | - | - | 19 | 13 | 12 | - | - | - | **3,022** |
| **ETO 0.25 µM** | 11 | 13 | 10 | - | - | - | 1 | 4 | 1 | - | - | - | **3,059** |
| **MMC 2.5 µM** | 9 | 8 | 6 | - | - | - | 2 | 4 | 6 | - | - | - | **3,069** |
| **EMS 0.5 mM** | - | - | - | 7 | - | - | - | - | - | 4 | - | - | **1,048** |
| **EMS 0.75 mM** | 7 | 8 | 7 | - | - | - | 4 | 3 | 3 | - | - | - | **3,022** |
| **6+0.5 h** | | | | | | | | | | | | | |
| **0.1 % DMSO** | - | 11 | 7 | 10 | 8 | 10 | - | 1 | 0 | 1 | 2 | 2 | **9,187** |
| **IRI 2 µM** | - | - | - | 9 | - | - | - | - | - | 3 | - | - | **1,004** |
| **IRI 4 µM** | - | - | - | 8 | 9 | 10 | - | - | - | 2 | 1 | 1 | **3,065** |
| **IRI 9 µM** | - | 6 | 6 | 6 | - | - | - | 1 | 3 | 3 | - | - | **4,022** |
| **IRI 17 µM** | - | 8 | 10 | 13 | - | - | - | 5 | 3 | 5 | - | - | **3,168** |
| **IRI 38 µM** | - | 14 | 9 | 9 | - | - | - | 9 | 10 | 8 | - | - | **6,226** |
| **IRI 66 µM** | - | 9 | 8 | 8 | - | - | - | 10 | 9 | 12 | - | - | **6,17** |
| **DES 15 µM** | - | - | - | - | 6 | 8 | - | - | - | - | 15 | 14 | **2,002** |
| **DES 25 µM** | - | 11 | 11 | 11 | - | - | - | 22 | 16 | 18 | - | - | **3,056** |
| **ETO 0.25 µM** | - | 10 | 12 | 7 | - | - | - | 1 | 3 | 3 | - | - | **3,019** |
| **MMC 2.5 µM** | - | - | - | - | - | - | - | - | - | - | - | - | **-** |
| **EMS 0.5 mM** | - | - | - | - | - | - | - | - | - | - | - | - | **-** |
| **EMS 0.75 mM** | - | - | - | - | - | - | - | - | - | - | - | - | **-** |

|  | **Micronucleated cells per 1,000 cells** | | | | | | | | | | | | **∑cells scored** |
| --- | --- | --- | --- | --- | --- | --- | --- | --- | --- | --- | --- | --- | --- |
|  | **MNC-** | | | | | | **MNC+** | | | | | |  |
|  | Experiment | | | | | | Experiment | | | | | |  |
|  | 1 | 2 | 3 | 4 | 5 | 6 | 1 | 2 | 3 | 4 | 5 | 6 |  |
| **6+1 h** | | | | | | | | | | | | | |
| **0.1 % DMSO** | - | 10 | 8 | 10 | 9 | 7 | - | 1 | 0 | 2 | 1 | 3 | **8,326** |
| **IRI 2 µM** | - | - | - | 9 | - | - | - | - | - | 4 | - | - | **1,014** |
| **IRI 4 µM** | - | - | - | 10 | 11 | 11 | - | - | - | 1 | 3 | 1 | **3,052** |
| **IRI 9 µM** | - | 8 | 5 | 17 | - | - | - | 0 | 1 | 1 | - | - | **3,065** |
| **IRI 17 µM** | - | 10 | 8 | 10 | - | - | - | 3 | 5 | 10 | - | - | **3,141** |
| **IRI 38 µM** | - | 14 | 6 | 11 | - | - | - | 8 | 7 | 15 | - | - | **5,113** |
| **IRI 66 µM** | - | 9 | 2 | 13 | - | - | - | 4 | 10 | 15 | - | - | **6,155** |
| **DES 15 µM** | - | - | - | - | 6 | 8 | - | - | - | - | 13 | 10 | **2,026** |
| **DES 25 µM** | - | 6 | 9 | 4 | - | - | - | 27 | 14 | 28 | - | - | **3,086** |
| **ETO 0.25 µM** | - | 11 | 16 | 11 | - | - | - | 6 | 3 | 4 | - | - | **3,018** |
| **MMC 2.5 µM** | - | - | - | - | - | - | - | - | - | - | - | - | **-** |
| **EMS 0.5 mM** | - | - | - | - | - | - | - | - | - | - | - | - | **-** |
| **EMS 0.8 mM** | - | - | - | - | - | - | - | - | - | - | - | - | **-** |
| **6+3 h** | | | | | | | | | | | | | |
| **0.1 % DMSO** | - | 9 | 12 | 11 | 14 | 9 | - | 0 | 1 | 1 | 1 | 5 | **9,23** |
| **IRI 2 µM** | - | - | - | 12 | - | - | - | - | - | 3 | - | - | **1** |
| **IRI 4 µM** | - | - | - | 9 | 13 | 10 | - | - | - | 1 | 1 | 3 | **3,027** |
| **IRI 9 µM** | - | 7 | 8 | 9 | - | - | - | 2 | 1 | 2 | - | - | **3,155** |
| **IRI 17 µM** | - | 8 | 8 | 7 | - | - | - | 3 | 4 | 8 | - | - | **3,147** |
| **IRI 38 µM** | - | 18 | 14 | 16 | - | - | - | 10 | 13 | 17 | - | - | **6,099** |
| **IRI 66 µM** | - | 15 | 14 | 12 | - | - | - | 9 | 12 | 19 | - | - | **6,093** |
| **DES 15 µM** | - | - | - | - | 5 | 4 | - | - | - | - | 32 | 21 | **2,075** |
| **DES 25 µM** | - | 10 | 8 | 2 | - | - | - | 19 | 33 | 32 | - | - | **3,161** |
| **ETO 0.25 µM** | - | 15 | 14 | 13 | - | - | - | 2 | 4 | 3 | - | - | **3,168** |
| **MMC 2.5 µM** | - | - | - | - | - | - | - | - | - | - | - | - | **-** |
| **EMS 0.5 mM** | - | - | - | - | - | - | - | - | - | - | - | - | **-** |
| **EMS 0.8 mM** | - | - | - | - | - | - | - | - | - | - | - | - | **-** |

|  | **Micronucleated cells per 1,000 cells** | | | | | | | | | | | | **∑cells scored** |
| --- | --- | --- | --- | --- | --- | --- | --- | --- | --- | --- | --- | --- | --- |
|  | **MNC-** | | | | | | **MNC+** | | | | | |  |
|  | Experiment | | | | | | Experiment | | | | | |  |
|  | 1 | 2 | 3 | 4 | 5 | 6 | 1 | 2 | 3 | 4 | 5 | 6 |  |
| **6+6 h** | | | | | | | | | | | | | |
| **0.1 % DMSO** | 4 | 10 | 7 | 14 | 7 | 7 | 1 | 1 | 1 | 1 | 3 | 3 | **12,191** |
| **IRI 2 µM** | - | - | - | 10 | - | - | - | - | - | 1 | - | - | **1,002** |
| **IRI 4 µM** | - | - | - | 11 | 12 | 9 | - | - | - | 1 | 1 | 5 | **3,047** |
| **IRI 9 µM** | - | 13 | 7 | 10 | - | - | - | 1 | 1 | 1 | - | - | **3,109** |
| **IRI 17 µM** | - | 8 | 8 | 20 | - | - | - | 4 | 1 | 7 | - | - | **3,084** |
| **IRI 38 µM** | 9 | 17 | 9 | 18 | - | - | 1 | 10 | 6 | 16 | - | - | **7,136** |
| **IRI 66 µM** | - | 21 | 10 | 16 | - | - | - | 15 | 9 | 14 | - | - | **5,133** |
| **DES 15 µM** | - | - | - | 5 | 7 | - | - | - | - | - | 17 | 9 | **2,042** |
| **DES 25 µM** | 11 | 6 | 10 | - | - | - | 32 | 32 | 26 | - | - | - | **3,089** |
| **ETO 0.25 µM** | 26 | 36 | 25 | - | - | - | 5 | 4 | 3 | - | - | - | **3,069** |
| **MMC 2.5 µM** | 17 | 11 | 10 | - | - | - | 2 | 3 | 3 | - | - | - | **3,046** |
| **EMS 0.5 mM** | - | - | - | 9 | - | - | - | - | - | 3 | - | - | **1,036** |
| **EMS 0.8 mM** | 19 | 11 | 10 | - | - | - | 1 | 4 | 1 | - | - | - | **3,096** |
| **6+15 h** | | | | | | | | | | | | | |
| **0.1 % DMSO** | 13 | 6 | 9 | 9 | 9 | 11 | 0 | 2 | 1 | 1 | 1 | 1 | **10,213** |
| **IRI 2 µM** | - | - | - | 13 | - | - | - | - | - | 1 | - | - | **1,091** |
| **IRI 4 µM** | - | - | 8 | 12 | 10 | - | - | - | 2 | 1 | 3 | - | **3,111** |
| **IRI 9 µM** | 12 | 14 | 8 | - | - | - | 1 | 0 | 3 | - | - | - | **3,039** |
| **IRI 17 µM** | 11 | 12 | 7 | - | - | 15 | 1 | 1 | 4 | - | - | 6 | **4,303** |
| **IRI 38 µM** | 14 | 14 | 17 | - | - | - | 2 | 4 | 6 | - | - | - | **6,225** |
| **IRI 66 µM** | 17 | 20 | 17 | - | - | - | 5 | 5 | 19 | - | - | - | **6,181** |
| **DES 15 µM** | - | - | - | 11 | 16 | - | - | - | - | 7 | 2 | - | **2,047** |
| **DES 25 µM** | 11 | 14 | 16 | - | - | 12 | 21 | 9 | 7 | - | - | 11 | **4,044** |
| **ETO 0.25 µM** | 19 | 53 | 40 | - | - | 66 | 4 | 4 | 1 | - | - | 3 | **4,093** |
| **MMC 2.5 µM** | 26 | 18 | 21 | - | - | - | 3 | 2 | 1 | - | - | - | **3,115** |
| **EMS 0.5 mM** | - | - | - | 13 | - | - | - | - | - | 1 | - | - | **1,007** |
| **EMS 0.8 mM** | 19 | 15 | 17 | - | - | - | 1 | 3 | 2 | - | - | - | **3,194** |

|  | **Micronucleated cells per 1,000 cells** | | | | | | | | | | | | **∑cells scored** |
| --- | --- | --- | --- | --- | --- | --- | --- | --- | --- | --- | --- | --- | --- |
|  | **MNC-** | | | | | | **MNC+** | | | | | |  |
|  | Experiment | | | | | | Experiment | | | | | |  |
|  | 1 | 2 | 3 | 4 | 5 | 6 | 1 | 2 | 3 | 4 | 5 | 6 |  |
| **6+24 h** | | | | | | | | | | | | | |
| **0.1 % DMSO** | 11 | 9 | 10 | 11 | 9 | 12 | 1 | 1 | 2 | 2 | 3 | 1 | **22,163** |
| **IRI 2 µM** | - | - | - | 10 | - | - | - | - | - | 1 | - | - | **1,035** |
| **IRI 4 µM** | - | - | - | 11 | 10 | 10 | - | - | - | 1 | 1 | 1 | **5,183** |
| **IRI 9 µM** | 9 | 11 | 11 | 10 | - | - | 0 | 0 | 1 | 2 | - | - | **9,222** |
| **IRI 17 µM** | 11 | 12 | 10 | 12 | - | - | 1 | 2 | 1 | 2 | - | - | **8,336** |
| **IRI 38 µM** | 14 | 14 | 17 | 25 | - | - | 1 | 1 | 2 | 3 | - | - | **18,755** |
| **IRI 66 µM** | 26 | 22 | 19 | 18 | - | - | 2 | 4 | 6 | 14 | - | - | **16,51** |
| **DES 15 µM** | - | - | - | 10 | 11 | - | - | - | - | 4 | 1 | - | **4,146** |
| **DES 25 µM** | 10 | 21 | 21 | - | - | - | 5 | 3 | 3 | - | - | - | **5,213** |
| **ETO 0.25 µM** | 28 | 49 | 38 | - | - | - | 2 | 3 | 1 | - | - | - | **5,208** |
| **MMC 2.5 µM** | 31 | 21 | 23 | - | - | - | 2 | 3 | 1 | - | - | - | **5,199** |
| **EMS 0.5 mM** | - | - | - | 26 | - | - | - | - | - | 1 | - | - | **2,164** |
| **EMS 0.8 mM** | 52 | 23 | 30 | - | - | 27 | 1 | 2 | 2 | - | - | 1 | **6,324** |
| **6+30 h** | | | | | | | | | | | | | |
| **0.1 % DMSO** | 10 | 11 | 11 | 10 | 12 | 10 | 1 | 0 | 2 | 1 | 2 | 3 | **10,769** |
| **IRI 2 µM** | - | - | - | 14 | - | - | - | - | - | 2 | - | - | **1,095** |
| **IRI 4 µM** | - | - | - | 9 | 9 | 13 | - | - | - | 1 | 1 | 1 | **3,143** |
| **IRI 9 µM** | 10 | 13 | 8 | 10 | - | - | 0 | 0 | 3 | 2 | - | - | **4,219** |
| **IRI 17 µM** | 10 | 11 | 17 | 17 | - | - | 0 | 3 | 1 | 2 | - | - | **6,079** |
| **IRI 38 µM** | 15 | 20 | 15 | 15 | - | - | 1 | 0 | 4 | 4 | - | - | **8,512** |
| **IRI 66 µM** | 21 | 16 | 12 | - | - | - | 2 | 3 | 1 | - | - | - | **6,193** |
| **DES 15 µM** | - | - | - | 17 | 15 | - | - | - | - | 1 | 3 | - | **2,031** |
| **DES 25 µM** | 15 | 10 | 11 | - | - | - | 4 | 2 | 6 | - | - | - | **3,176** |
| **ETO 0.25 µM** | 15 | 33 | 21 | - | - | - | 2 | 4 | 2 | - | - | - | **3,17** |
| **MMC 2.5 µM** | 31 | 24 | 27 | - | - | - | 4 | 3 | 1 | - | - | - | **3,127** |
| **EMS 0.5 mM** | - | - | - | 43 | - | - | - | - | - | 3 | - | - | **1,037** |
| **EMS 0.8 mM** | 39 | 31 | 33 | - | - | - | 1 | 1 | 2 | - | - | - | **3,107** |

|  | **Micronucleated cells per 1,000 cells** | | | | | | | | | | | | **Total number of cells scored** |
| --- | --- | --- | --- | --- | --- | --- | --- | --- | --- | --- | --- | --- | --- |
|  | **MNC-** | | | | | | **MNC+** | | | | | |  |
|  | Experiment | | | | | | Experiment | | | | | |  |
|  | 1 | 2 | 3 | 4 | 5 | 6 | 1 | 2 | 3 | 4 | 5 | 6 |  |
| **6+40 h** | | | | | | | | | | | | | |
| **0.1 % DMSO** | 9 | 14 | 13 | 9 | 10 | 9 | 1 | 3 | 2 | 3 | 1 | 1 | **10,328** |
| **IRI 2 µM** | - | - | - | 12 | - | - | - | - | - | 1 | - | - | **1,059** |
| **IRI 4 µM** | - | - | 11 | 8 | 18 |  | - | - | 2 | 4 | 3 |  | **3,104** |
| **IRI 9 µM** | 9 | 13 | 14 | - | - | 9 | 2 | 1 | 1 | - | - | 1 | **4,087** |
| **IRI 17 µM** | 11 | 11 | 15 | - | - |  | 1 | 0 | 2 | - | - | - | **3,09** |
| **IRI 38 µM** | 13 | 13 | 40 | - | - |  | 0 | 4 | 8 | - | - | - | **6,208** |
| **IRI 66 µM** | 15 | 21 | 28 | - | - |  | 2 | 6 | 7 | - | - | - | **6,425** |
| **DES 15 µM** | - | - | - | 13 | 10 |  | - | - | - | 1 | 1 | - | **2,004** |
| **DES 25 µM** | 18 | 18 | 18 | - | - |  | 3 | 3 | 2 | - | - | - | **3,127** |
| **ETO 0.25 µM** | 30 | 38 | 38 | - | - |  | 3 | 2 | 2 | - | - | - | **3,17** |
| **MMC 2.5 µM** | 30 | 36 | 25 | - | - |  | 4 | 4 | 1 | - | - | - | **3,147** |
| **EMS 0.5 mM** | - | - | - | 36 | - |  | - | - | - | 2 | - | - | **1,009** |
| **EMS 0.8 mM** | 53 | 46 | 56 | - | - |  | 3 | 3 | 3 | - | - | - | **3,086** |
